# Use of Biopowders as Adsorbents of Potentially Toxic Elements Present in Aqueous Solutions

**DOI:** 10.3390/ma18030625

**Published:** 2025-01-30

**Authors:** Vanesa Santás-Miguel, Vanesa Lalín-Pousa, Manuel Conde-Cid, Andrés Rodríguez-Seijo, Paula Pérez-Rodríguez

**Affiliations:** 1 Department of Plant Biology and Soil Science, Area of Soil Science and Agricultural Chemistry, Faculty of Sciences, University of Vigo, 32004 Ourense, Spain; vsantas@uvigo.es (V.S.-M.); vanesa.lalin@uvigo.gal (V.L.-P.); manconde@uvigo.gal (M.C.-C.); andresrodriguezseijo@uvigo.gal (A.R.-S.); 2 Agroecology and Food Institute (IAA), University of Vigo—Campus Auga, 32004 Ourense, Spain; 3 Microbial Ecology, Department of Biology, Lund University, Ecology Building, 22362 Lund, Sweden

**Keywords:** cork bark, pine bark, water pollution, arsenic, chromium, phosphorous, fluoride

## Abstract

This study examines the adsorption and desorption behaviors of phosphorus (P), arsenic (As), fluoride (F), and chromium (Cr) in aqueous solutions on green materials such as cork bark (CB) and pine bark (PB). These materials are characterized by active functional groups and net negative charges on their surfaces and porous structures. The evaluation considers variations in contaminant concentrations (0.01–10 mM) and pH (3.5–12). Cork bark exhibited higher adsorption capacity for As and F, while PB was more effective for P and Cr. Adsorption isotherms followed the Freundlich and Langmuir models, indicating surface heterogeneity and multilayer adsorption for most potentially toxic elements (PTEs). Desorption tests demonstrated low rates, with CB retaining up to 99% of F and 85% of As, and PB achieving up to 86% retention for Cr and 70% for P. The influence of pH was minimal for As, P, and F, but acidic conditions significantly enhanced Cr adsorption, showing similar behavior for both biopowders. These findings suggest that CB and PB biopowders are promising, environmentally friendly biosorbents for the removal of PTEs from aqueous solutions. Their effectiveness varies depending on the specific contaminant. This study highlights the potential of these natural materials for sustainable applications in water treatment and soil remediation.

## 1. Introduction

Potentially toxic elements (PTEs) are found in nature, usually present at levels less than 0.1%, but at abnormally higher concentrations, they can become harmful, with detrimental effects on the ecological balance of the environment and human health [1,2,3]. Alterations in high amounts are caused by various anthropogenic activities, such as industrial activities, agricultural exploitation with the use of chemicals, industrial activities, or sectors like microelectronics, among others [4]. Phosphorous, As, F, and Cr are good examples of PTEs that occur very frequently in the environment, usually in the forms P-PO_4_^−3^, As(V), F^−^, and Cr(VI) in solution, so their study is of great interest to avoid detrimental damage. Phosphorus is an essential macronutrient for plants, soil organisms, and humans. In the case of plants, its functions cannot be performed by any other element, and an adequate supplement of P is needed for optimal growth and reproduction. Specifically, P is usually absorbed by plants as H_2_PO_4_^−^ or HPO_4_^2−^, depending on whether the predominant soil pH is acidic or alkaline, respectively. To ensure proper growth, phosphorus is provided as a fertilizer in agriculture as an external input. However, excessive doses can lead to its leaching and, consequently, to water pollution and eutrophication [5], especially in overfertilized soils. Additionally, P-based compounds, such as organophosphorus compounds, are a broad class of chemicals widely used mainly in agriculture as pesticides; in industry as plasticizers, flame retardants, and lubricants; and as pharmaceuticals, such as certain antiparasitic drugs. Despite organophosphorus compounds tending to degrade relatively quickly, their residues may disrupt the natural balance of soil microorganisms, affecting nutrient cycling, soil fertility, and plant growth. For instance, P (as well as As) organometallic compounds can deposit as metallic P (and As) by oxidation, which can result in hazardous effects [6]. Apart from affecting soil health, they may contaminate water bodies and can be ecotoxic to a wide range of organisms [7,8]. Acute and chronic human exposure can lead to severe health effects, necessitating careful management and regulation. Although normal P-Olsen concentrations in the soil range from 19 to 50 mg/kg to maintain agronomic and environmental thresholds that optimize agricultural nutrition and minimize P losses [9], the P concentration must be significantly reduced in waters in order to maintain a good ecological status. For instance, concentrations discharged by wastewater discharges include a total phosphorus limit of 0.5 mg/L or 90% phosphorus removal for wastewater treatment plants [10]. Although there is no established limit for P in surface or drinking water by the European Water Framework Directive and the U.S. EPA, respectively, Canadian regulations establish that levels of total phosphorus should generally remain below 0.03 mg/L in streams and below 0.01 mg/L in lakes to prevent excessive algal growth and maintain good ecological status [11]. Therefore, maintaining low phosphorus levels in various water bodies is crucial to safeguard aquatic ecosystems and human health.

Arsenic is widely distributed in the environment, being found not only in mines but also in the air, water, and plants that adsorb it and, thus, in food, acting as a human carcinogen element [12,13,14]. Its toxicity depends on the chemical species, with inorganic species being more toxic than organic species, and As(III) being more toxic than As(V). Arsenic can be found in a large number of minerals, ranging in soil between 10 and 50 mg/kg and being phytotoxic depending on the type of soil [15,16]. For plants, low arsenic concentrations may stimulate plant growth, despite its non-essentiality. However, As participates in a series of metabolic processes in plants that can inhibit their growth and even lead to plant death at high concentrations [17]. Toxic effects in the plant are considered to occur when concentrations exceed 2–10 mg/kg [18]. This element is used in agriculture, pesticides, coal combustion, and mining, so these are the main anthropogenic activities that emit As into soils [19]. Regarding groundwater, the World Health Organization (WHO) has set the limit for arsenic in drinking water at 10 µg/L [20].

Fluoride in water is a pollutant of high environmental and public health concern, particularly in regions where natural or anthropogenic factors contribute to elevated fluoride levels. While fluoride is naturally present in groundwater and some surface waters due to the weathering of fluoride-containing minerals like fluorite, cryolite, and apatite, it can also be introduced by human activities such as industrial processes, the use of phosphate fertilizers, and, in some cases, coal burning. The maximum allowable fluoride concentration in drinking water is 1.5 mg/L according to the WHO and the EU [20]. Concentrations above this level are considered hazardous due to the risk of fluorosis, particularly with long-term exposure.

Hexavalent chromium, Cr(VI), is highly toxic due to its mutagenic, carcinogenic, and teratogenic potential. Chromium has no essential metabolic function in plants and can cause numerous physiological, morphological, and biochemical toxic effects in excessive amounts. Hexavalent chromium is highly soluble, so its mobility in soil and aquatic environments is easy, and consequently, it causes toxicity and contamination in ecosystems [21]. Human activities such as industrial discharges, mining activities, waste disposal, and coal combustion and its presence in fertilizers and pesticides have significantly contributed to the release of chromium, particularly Cr(VI), into the environment. The WHO and the EU have set a limit of 0.05 mg/L for total chromium in drinking water [20].

Cork bark (CB) biopowder is a finely milled byproduct derived from the outer layer of cork oak trees (*Quercus suber* L.). It is predominantly used in the food industry for manufacturing bottle stoppers and as activated carbon. Additionally, it has potential applications in the pharmacological industry [22]. During industrial transformation processes, substantial quantities of cork biopowder are produced because of grinding, cutting, and finishing operations [23]. A small fraction of this material is reused within the cork stopper manufacturing industry for its refinement. However, the excess that is not required for this purpose is discarded and must undergo appropriate treatment to prevent environmental accumulation and potential contamination. This material has been identified as a highly effective biosorbent for various pollutants, including heavy metals, oils, pesticides, and volatile organic compounds (VOCs) in water systems [24,25,26,27]. More recently, [28] tested its use as a biosorbent for potentially toxic elements, demonstrating that CB exhibits a strong retention capacity for Fe and Cd in aqueous solutions under acidic conditions. Additionally, at higher pH levels, cork powder was more efficient in adsorbing Zn. Furthermore, emerging contaminants, such as antibiotics, have been effectively removed using cork powder, showing remarkably high adsorption capacities [29]. Therefore, CB is a green, renewable, and natural material with a porous structure and active functional groups (Supplementary Material Table S2, Figure S2), making it an attractive material for adsorption processes [21,27]. In addition, until now, it has never been tested without any pretreatment to specifically adsorb As, F, P, and Cr as pollutants in aqueous solutions.

Pine bark is obtained from the *Pinus Pinaster* species, an evergreen tree noted for its rapid growth and long life. The global production of pine wood gives rise to a large amount of generated pine bark, and due to its large volume and the large quantities produced, it is very important to manage it properly, which, in turn, generates additional costs associated with having it as waste. Its use as a biosorbent to immobilize pollutants is a good alternative within the circular economy to reduce costs while providing sustainable environmental solutions. Pine bark biopowder has been successfully tested in soils and aqueous solutions as raw material [28,30,31] and chemically modified [32] to immobilize PTEs such as Fe, Cd, Cu, Ni, Pb, and Zn, as well as emerging pollutants such as antibiotics in order to improve soil microbiology [29,33,34]. Similarly to CB, PB has not been tested before without chemical modification to adsorb the target PTEs. Functional groups on its surface are shown in Table S2 and Figure S2 (Supplementary Material).

As conditions in nature may change very quickly due to non-point source pollution, which can provide a wide range of inputs leading to changing microscale physicochemical properties, it is important to test the potential adsorbent properties of these biopowders under different conditions. Therefore, the aim of this study is to evaluate the potential of cork bark and pine bark biopowders, naturally sourced without any pretreatment, as adsorbents for the removal of specific and previously untested potentially toxic elements (PTEs). This study considers their distinct and inherent physicochemical properties under a wide range of environmental conditions. Specifically, P, As, F, and Cr are used as PTEs considering a wide range of pH (3.5–12) and concentrations for aqueous solutions (0.01–10 mM). As all these PTEs behave as anions in solutions, a comparative evaluation of these biopowders will provide information on their efficiency, suitability, and applicability as adsorbents of anionic PTEs in aqueous solutions, based on the hypothesis that both materials are suitable as biosorbents for all the studied PTEs. We additionally hypothesize that both biopowders may thus contribute to the development of sustainable environmental remediation strategies, reducing the environmental risk of the studied PTEs. With the results of this study, it is expected that the potential adsorption of cork bark and pine bark biopowders for PTEs will be revealed.

## 2. Material and Methods

### 2.1. Biopowders Used

The biopowders used were PB and CB supplied by Geolia (Madrid, Spain) and Corchos Almeida S.L. (Ourense, Spain), respectively. Both materials were used in their natural forms, without chemical pretreatment. The pine bark was pre-shredded to powder size, and the cork powder was used directly as supplied by the industry in powder size. The physicochemical properties of both biopowders were measured previously and are shown in González-Feijoo et al. (2024) [28]. They are summarized in Table 1, and their Z potential is shown in Figure S1. Additionally, microphotographs of both biopowders obtained by SEM for PB and CB have been previously provided to visualize their structures [21,35,36].

The negative zeta potential of pine bark and cork bark biopowders at almost all pH levels is primarily due to the abundance of ionizable phenolic, carboxylic, hydroxyl, and carbonyl groups on their surfaces (Table S2 and Figure S2), coupled with their chemical composition (e.g., lignin, holocellulose and hemicellulose, and polysaccharides, Table 1). These functional groups readily deprotonate and contribute to a stable negative surface charge over a broad pH range [28,37].

### 2.2. Adsorption–Desorption Experiments

Firstly, adsorption experiments as a function of time (adsorption kinetics) were previously carried out by González-Feijoo et al. (2024) [28] for CB and by Cutillas-Barreiro et al. (2014) [30] for PB to check the adsorption time required to reach equilibrium. The results for both biopowders showed that adsorption took place rapidly from 1 to 48 h, without significant differences among the studied elements. Twenty-four hours was the shortest time in which equilibrium was reached for all the studied elements for both materials. Therefore, the chosen time to perform the experiments was 24 h.

Secondly, the adsorption (1)–desorption (2) behavior at equilibrium for both biopowders was studied through batch-type experiments by testing the pollutants’ concentrations.

(1) To test the adsorption behavior at equilibrium as a function of the pollutant’s concentration, 0.25 g of each biopowder (CB and PB) was placed in a 50 mL Falcon tube, and 25 mL of solutions containing different concentrations of the studied PTEs was added, each one individually. These elements were added as salts (provided by Panreac Química S.L.U., Barcelona, Spain) from NaH_2_PO_4_ for P, Na_2_HAsO_4_ for As, K_2_Cr_2_O_7_ for Cr, and KF for F. Each element was added to each biopowder at different concentrations of 0 (control), 0.01, 0.025, 0.05, 0.1, 0.25, 0.5, 1, 2.5, 5, and 10 mM in NaNO_3_ 0.01 M (Panreac Química S.L.U., Barcelona, Spain) as a background solution for As, Cr, F, and P. The resulting suspensions (biopowder + each PTE) were shaken for 24 h, centrifuged (4000 rpm, 15 min), and filtered to obtain the liquid extracts, where pH was determined. The concentrations of P were measured by spectrophotometry, while As, F, and Cr were measured by Inductively Coupled Plasma–Mass Spectrometry (ICP-MS, Thermo Elemental, Cetac ASX-520 Autosampler, ThermoFisher Scientific, Waltham, MA, USA).

(2) Desorption experiments were carried out on the samples resulting from the previous adsorption tests by adding 25 mL of NaNO_3_ 0.01 M. Then, the samples were shaken for 24 h, centrifuged, and filtered, and P, As, F, and Cr, as well as pH, were determined in the equilibrium solutions following the same previous protocols.

### 2.3. Adsorption Experiments with Varying pH

A second set of experiments was conducted to evaluate the adsorption behavior as a function of solution pH. This was achieved by adjusting the pH with increasing volumes of 0.5 M NaOH (Panreac Química S.L.U., Barcelona, Spain) (0, 0.1, 0.2, 0.3, 0.4, 0.5, 0.6, 0.7, 0.8, 0.9, 1.0, 1.2, and 1.6 mL; final volume = 25 mL), resulting in a pH range of 3.5–12, representative of possible natural environmental conditions. A fixed concentration of 1 mM for each PTE (P, As, F, and Cr) was added to each sample following the same procedure as in the first experiment. The suspensions were shaken for 24 h, centrifuged at 4000 rpm for 15 min, and filtered. The pH and PTE concentrations in the equilibrium solutions were then measured using the same protocols as before. All experiments were performed in triplicate.

### 2.4. Data Treatment

Adsorption curves at equilibrium were fitted to the Freundlich (Equation (1)) and Langmuir (Equation (2)) models, as previously referenced [38]:

(1) $$C_{ads}=K_{F}\;C_{e}^{n}$$

(2) $$C_{ads}=\frac{K_{L}{XmC}_{e}}{1+K_{L}C_{e}}$$

*C_ads_* represents the amount of PTE adsorbed (mmol kg^−1^) at equilibrium, while *C_e_* denotes the concentration of PTE remaining in the solution at equilibrium (mM). $K_{F}$ is the Freundlich affinity coefficient (L*^n^* mmol^1−*n*^ kg^−1^), and *n* is the Freundlich linearity index (Equation (1)). For the Langmuir model,${\;K}_{L}$ is a Langmuir parameter related to the adsorption energy (L mmol^−1^), and *Xm* is the Langmuir maximum adsorption capacity (mmol kg^−1^) (Equation (2)).

Desorption was quantified as the amount of PTE desorbed (mmol kg^−1^) relative to the previously adsorbed amount. Adsorption experiment results at various PTE concentrations were analyzed using these equations with IBM SPSS v25 software.

## 3. Results and Discussion

### 3.1. The Adsorption–Desorption of PTEs by the Biopowders

**Figure 1:**
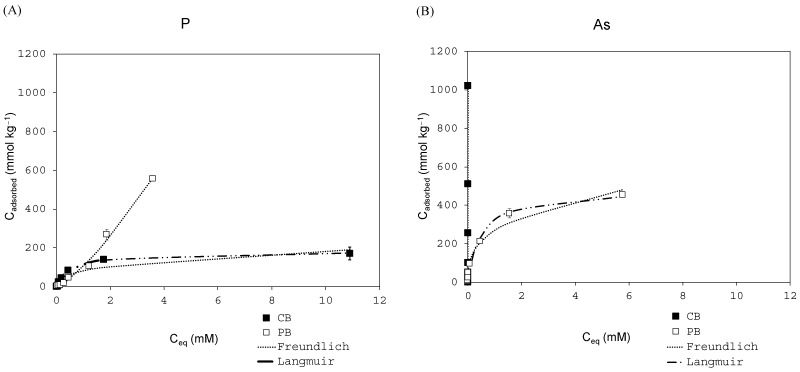

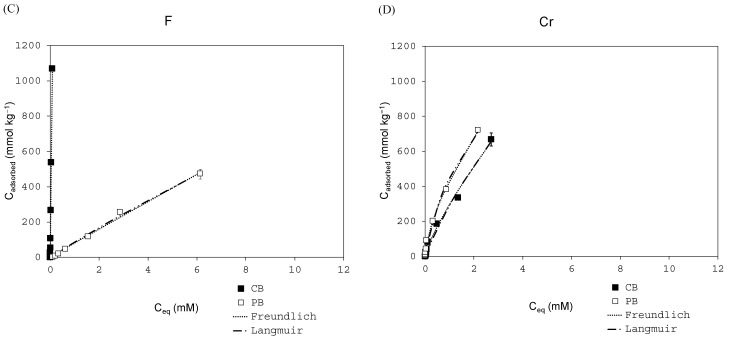
Adsorption curves for P (**A**), As (**B**), F (**C**), and Cr (**D**) as a function of the equilibrium concentration (C_eq_) obtained with cork bark (black squares) and pine bark (white squares). Average values for three replicates, with bars corresponding to standard deviations (standard deviations smaller than the icon size might not be visible).

Figure 1 shows the adsorption curves obtained for the four PTEs in the two biopowders. As observed, different types of adsorption curves were obtained depending on both the PTE and the biopowder tested. Thus, according to the classification proposed by Giles et al. (1974) [39], Giles Type-L curves were obtained for the adsorption of P in PB (Figure 1A), for the adsorption of As in PB (Figure 1B), and for the adsorption of Cr in both biopowders (CB and PB) (Figure 1D). On the other hand, Giles Type-C curves were obtained for the adsorption of As in CB (Figure 1B) and for F in both biopowders (Figure 1C), while a Giles Type-S curve was obtained for the adsorption of P in PB (Figure 1A).

Type-L curves indicate strong adsorbate–adsorbent interactions, leading to monolayer adsorption, with a sharp increase followed by a plateau when the monolayer is complete. As can be seen in Figure 1, a plateau is reached (Figure 1A,B) or is about to be reached (Figure 1D) while increasing the concentrations of P in CB (Figure 1A), of As in PB (Figure 1B), and of Cr in both biosorbents (Figure 1D). In addition, for the adsorption of As using PB as the sorbent, the behavior corresponds to a Type-H curve (Figure 1B), which is a special case of an L-Type curve, which occurs when the absorbent surface possesses a very strong affinity for the adsorbate [40]. The case of P adsorption with PB shows weak interactions between the adsorbent and adsorbate (Type-S curve characteristic), leading to slow initial adsorption and continuous multilayer adsorption with no clear saturation point (Figure 1A). This behavior may be favored by cells formed in the radial position of cork, together with the ionic species of P [22]. In the case of As and F with CB (Figure 1B,C), the adsorption is so high and quick as the concentration increases that the adsorption is still in its initial phase: i.e., CB still has adsorption capacity for higher concentrations. Finally, the case of F adsorption using PB resulted in a constant partition of the solute between the solution and the biosorbent (Type-C curve characteristic) [40].

Figure 1A shows the adsorption of P using both biopowders. Pine bark exhibited higher adsorption than CB, reaching 556 mmol/kg, while CB only reached 171 mmol/kg. In this case, P adsorption in PB followed an exponential trajectory, showing a concave shape at the beginning followed by a steep rise, and then leveled off at higher concentrations, suggesting that adsorption is less favorable at low concentrations but becomes significantly more favorable as the concentration increases (Type-S curve), up to a C_eq_ of 4 mM, which is indicative of weaker adsorption in the first layer. In the case of CB, adsorption increases exponentially at equilibrium concentrations <4 mM and tends to decrease at higher equilibrium concentrations. Regarding As adsorption (Figure 1B), both biopowders showed clearly different adsorption capacities, since PB showed exponential adsorption at the beginning, reaching stability after equilibrium concentrations ≈ 6 mM and a maximum As adsorption of 455 mmol/kg. On the other hand, CB showed a higher adsorption capacity at low equilibrium concentrations (<0.02 mM), reaching 1022 mmol/kg of adsorbed As. This implies that CB capacity for As adsorption is still incipient and the adsorption is not dependent on the initial concentration added in the range of tested concentrations (Type-C curve) [39]. The same behavior occurred in the case of F using both biopowders (Figure 1C), but it can be observed that the adsorption process is still in the initial phase since stabilization is not reached within the range of the studied concentrations. Despite that, CB resulted in a higher adsorption capacity than PB (1068 vs. 471 mmol/kg, respectively). Regarding Cr (Figure 1D), similar behavior was observed with both biopowders, although PB reached 720 mmol/kg versus 668 mmol/kg reached with CB. In summary, CB worked better for As and F, reaching higher adsorption than PB. Specifically, for CB, the following sequence was observed: F > As >> Cr > P. On the contrary, the adsorption of P and Cr worked better with PB, especially for P. In this case, PB showed the following adsorption sequence: Cr > P > F ≈ As. Similar results, although slightly lower, were obtained for Cr and F by Romar-Gasalla et al. (2018) [41] when using PB with up to 6 mM of added concentration. They found adsorption rates >97% and 62–73%, respectively, while in our study, the adsorption percentages were >80% and 22–48% for Cr and F, respectively. Regarding P, Yeager (1982) [42] obtained 0.3 mmol/kg maximum adsorption, but their maximum added concentration was 0.5 mM, around 50-fold lower than that obtained in our study. Paradelo et al. (2017) [43] found a low retention capacity of PB for phosphate, arsenate, and fluoride, while it was higher for dichromate. However, they performed different types of experiments (column experiments) and used lower added concentrations (generally 2.5 mM). The use of cork wastes was also tested for Cr(VI) in higher concentration ranges than those in our study, showing around half the maximum adsorption (≈300 mmol/kg) [26]. However, although cork bark has been tested to remove a wide range of pollutants in water [44], no other studies were found in the literature using CB with the rest of the PTEs tested in this study. Studies were only found for the removal of As and P using iron-coated cork granulates, obtaining satisfactory results, especially when increasing ionic strength and at high pH [45,46,47]. So, to the best of our knowledge, this is the first study where a natural biopowder of CB is tested as an adsorbent for P, As, and F (anionic form in solution). All these results have been summarized and compared with the adsorption obtained from other carbon-based materials (Table 2) to highlight the advantages of our work.

**Figure 2:**
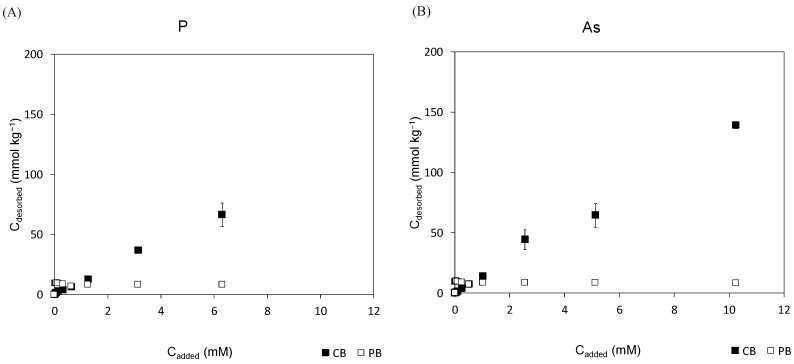

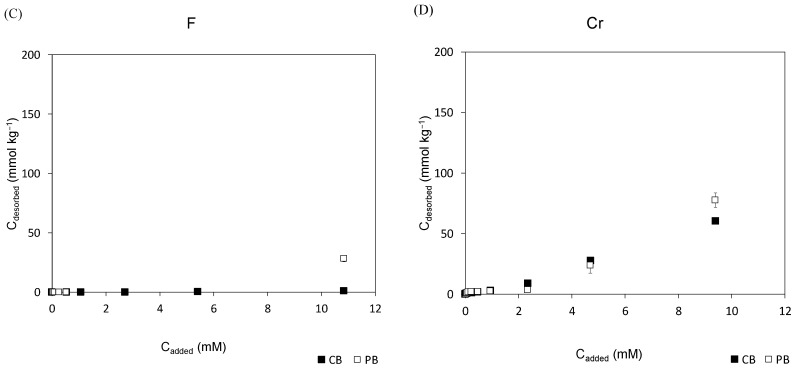
Desorption of P (**A**), As (**B**), F (**C**), and Cr (**D**) as a function of the added concentration (0–10 mM) of each of these elements to cork bark (black squares) and pine bark (white squares) biopowders. Average values for three replicates, with bars corresponding to standard deviations (standard deviations smaller than the icon size might not be visible). Note that the *Y*-axis has been reduced to better visualize the desorption behavior.

The results from desorption experiments are shown in Figure 2. The concentrations desorbed were very low as regards the added concentrations, although the trends increased with increasing added concentrations, especially with CB for As and P. However, it was observed that desorption was very low for all PTEs in both biopowders, especially at added concentrations <5 mM, at which desorption tended to increase.

In the case of CB, desorption was slightly higher than in PB for P and As, although it did not exceed 150 mmol/kg (specifically 66.4 and 168.8 mmol/kg, respectively). On the other hand, desorption was higher in PB than in CB for F and Cr, reaching 28.1 and 77.5 mmol/kg, respectively.

In the case of cork bark, the maximum desorption was higher for As > P ≈ Cr > F, with ranges between 1.2 and 138.8 mmol/kg. For PB, the maximum desorption was as follows: Cr > F > As ≈ P, with all being lower than 80 mmol/kg, specifically between 8.2 and 77.4 mmol/kg. The generally low desorption rates found with all studied PTEs using both biopowders indicated that the adsorption process was quite irreversible. The desorption weaknesses could be due to several reasons, such as strong adsorbate–adsorbent interactions due to ionic and covalent bonds; high surface heterogeneity of the adsorbent, with a high number of active sites, which results in the adsorbent exhibiting higher affinity for the adsorbate; ionic strength and pH, which may stabilize adsorbed ions through electrostatic interactions, reducing their likelihood of desorbing; or competitive adsorption of other ions or molecules present in the solution that may compete with the desorbed element ions, reducing the effectiveness of the desorption process; among others. This fact may contribute to the high retention capacity of these materials. Indeed, maximum retention capacity rates showed values between 57 and 99% for CB, while values between 47 and 86% were found for PB (Table 3), depending on the PTE studied. As a result, CB was more efficient for As and F retention, while PB was better for P and Cr. However, the retention of Cr was similar with both studied biopowders. These results indicate that these biopowders can be used as biosorbents of these PTEs in aqueous solutions at low pollutant concentrations. Similar results were previously obtained [24], where it was observed that PB and CB worked well as biosorbents for Fe and Cd, with PB exhibiting slightly better results, especially under a broader range of conditions. Furthermore, the lowest maximum adsorption capacity obtained for P using CB was almost the same as that obtained for Pb and similar to that for Zn, while the highest obtained for F was similar to that for Fe. Conversely, when using PB, the maximum retention capacity obtained for F was the lowest compared to that obtained for Fe, Cu, Zn, Cd, Ni, and Pb, but the opposite was true for Sn, which did not work at all. However, the percentages obtained for Cr were similar to those for Fe and Cd [28]. In this study, CB obtained a higher retention capacity than PB for the most retained elements. Overall, these results suggest that PB is more appropriate as a biosorbent for PTEs present in aqueous solutions in cationic form, while CB is more appropriate for elements in anionic form. The surface morphology of both materials significantly influences their capacity to retain PTEs, mainly affected by their surface area, porosity, functional groups, and surface roughness. For instance, CB has a higher surface area than PB due to its honeycomb-like structure and high porosity, while PB often has a rough and uneven surface, which also contributes to increasing its surface area, enhancing its ability to adsorb PTEs [51,52]. In addition, CB is rich in compounds such as suberin, lignin, and polysaccharides, while PB contains tannins, lignin, and cellulose. All these functional groups can provide active binding sites capable of binding PTEs [37].

### 3.2. Adsorption Isotherms

Table 4 shows the parameters obtained from the fitting of adsorption curves to the Freundlich and Langmuir models for all PTEs and for the two bioadsorbent materials. On the one hand, the Freundlich model satisfactorily described all the adsorption curves obtained, judging by the R^2^ values obtained, which ranged between 0.881 and 1.000 (Table 4). On the other hand, the Langmuir model satisfactorily described all the adsorption curves, with R^2^ values ranging between 0.986 and 0.994 (Table 4), except for the adsorption of As and F in CB and for the adsorption of P in PB, where the experimental data did not fit this model.

Data obtained from the Freundlich model show the highest *K_F_* values for As adsorption in CB and Cr adsorption in PB. This parameter is related to the multilayer adsorption capacity of a given adsorbent, which is consistent with observations previously made from the adsorption curves for these two specific elements and sorbents and is also indicative of higher adsorption. The lowest *K_F_* values were obtained for P using CB and F using PB sorbents. However, these *K_F_* values were slightly lower than those obtained by Quintáns-Fondo et al. (2019) [53] for Cr(VI) and F in simple systems using pine bark as the sorbent, although they followed the same trend of being higher for Cr(VI) than for F. In addition, *K_F_* was significantly correlated with the pH value in the adsorption process (r = −0.401, *p <* 0.0001), with *K_F_* increasing at lower pH (Table 5). Similar results were also obtained by González-Feijoo et al. (2024) [28] for cationic PTEs in the same type of sorbents, while Cela-Dablanca et al. (2022) [38] found a significant but positive correlation with pH for As adsorption in different soils and sorbent materials, including pine bark.

The *n* parameter provides insights into the intensity or favorability of the adsorption process, and it describes the heterogeneity of the adsorption surface. If *n* > 1, adsorption is favorable, meaning the process is more likely to occur, especially at lower solute concentrations. In this case, the surface is heterogeneous, and stronger adsorption sites are filled first. If *n* = 1, adsorption is linear, implying that the adsorption sites have equal affinity for the adsorbate at all concentrations. This scenario usually occurs when the adsorbent surface is homogeneous. Finally, if *n* < 1, adsorption is considered unfavorable, meaning it becomes more difficult as the concentration of the adsorbate increases [54,55]. As can be seen in Table 4, the *n* parameter is positive in all cases. In addition, *n* is nearer to 0 than 1 for P and As using CB and PB, respectively, meaning that, in these cases, adsorption is less favorable, especially at higher concentrations. The obtained *n* values using PB were lower than those obtained by Quintáns-Fondo et al. (2019) [53] and by Romar-Gasalla et al. (2018) [41] for Cr and for As [38]. This could be due to lower added concentrations (up to 6 mM for Cr and up to 1 mM for As) than those used in this study (up to 10 mM), which could allow for more favorable adsorption. Despite the *n* values being slightly higher than those obtained for F in simple systems using PB, their R^2^ values were quite similar [41]. It should be highlighted that the results showed *n* ≈ 1 in the adsorption curves of F using both studied biopowders, indicating linear adsorption without differences in affinity regarding concentrations. Contrarily, *n* > 1 was indicated for P using PB, resulting in more favorable adsorption, which is in line with the obtained curve suggesting multilayer adsorption. Generally, the most favorable adsorption was slightly better with PB than with CB for the studied PTEs.

Regarding the Langmuir model, the *K_L_* parameter represents the affinity between the adsorbate and the adsorbent. A higher *K_L_* value suggests a stronger interaction between the adsorbate and the adsorbent surface. Very high values were obtained for As adsorption on PB, followed by P on PB, while weaker binding energy resulted for Cr and F on CB and PB, respectively. Considering PB as a biosorbent, low values were obtained for *K_L_*, with higher values for As, followed by Cr > F. Our results are slightly different from those obtained by Cela-Dablanca et al. (2022) [38] for As and by Romar-Gasalla et al. (2018) [41] and Quintáns-Fondo et al. (2019) [53] for Cr and F using PB, although all of them were low. However, in the case of CB, *K_L_* values were similar to those obtained by Fiol et al. (2003) [26] for Cr, while they were completely different from those obtained by Almeida (2015) [25], although she subjected the CB to a previous treatment.

The parameter *Xm* of the Langmuir equation represents the maximum adsorption capacity of the adsorbent, which is the highest amount of adsorbate that can be adsorbed per unit mass of adsorbent when all the available adsorption sites are fully occupied [54,56]. The highest *Xm* values for CB biopowder were obtained for Cr, followed by P, while for PB biopowder, the highest *Xm* values were obtained for F, followed by Cr > As. It should be highlighted that *Xm* values were generally higher for PB than for CB, considering the adjusted parameters. Studies by Romar-Gasalla et al. (2018) [41] and Quintáns-Fondo et al. (2019) [53] obtained much lower values for F, while their data could not be well fitted to this model for Cr. Regarding As, we obtained lower *Xm* values than Cela-Dablanca et al. (2022) [38], while the opposite was true for those obtained by Quintáns-Fondo et al. (2019) [53] using PB. Overall, CB showed a higher *Xm* for Cr, while it was higher for F using PB.

### 3.3. PTE Adsorption as a Function of pH

**Figure 3:**
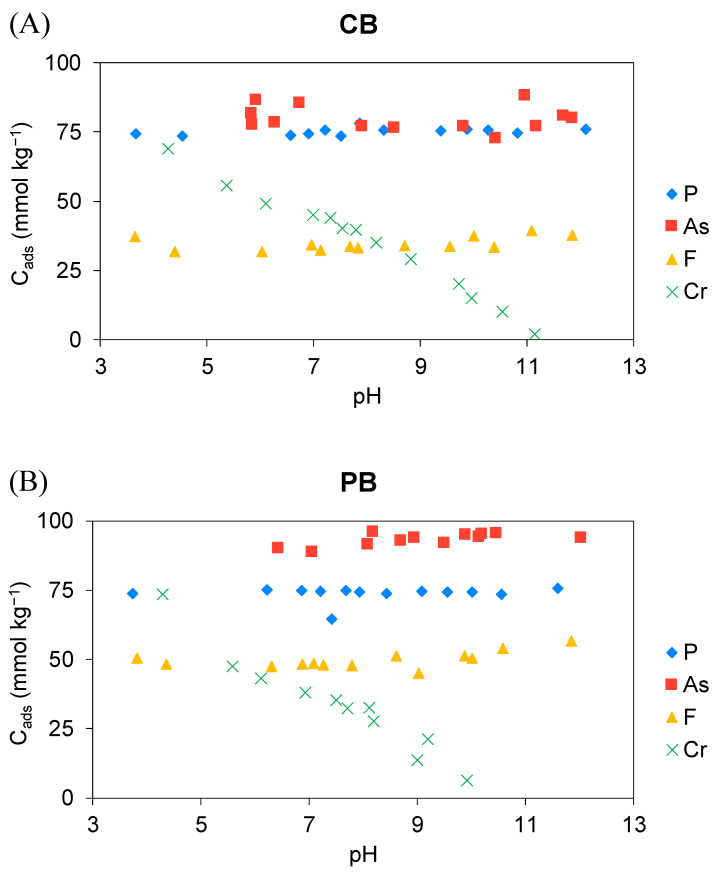
Adsorbed concentrations of the studied PTEs as a function of pH solution on (**A**) cork bark (CB) and (**B**) pine bark (PB) biopowders.

The effect of the pH solution on PTE adsorption by the two biopowders is shown in Figure 3. As can be seen in Figure 3A, P and F adsorption is less affected by pH, resulting in almost constant adsorption on CB in the studied range, especially for P. Fluoride showed a slight increase in adsorption with increasing pH, although this was not significant, with the lowest adsorption at pH ≈ 4. Regarding As, the highest adsorption on CB was obtained at pH 11, followed by pH 6 and 7. Although differences with other pHs were not huge, adsorption concentrations were >72 mmol/kg in all cases. Contrarily, Cr adsorption showed a clear dependence on pH regarding its adsorption on CB, being higher at acidic pH and tending to decrease with increasing pH. In the studied range of pH, higher adsorption on CB was obtained for As > P > Cr > F.

Similar trends were observed when using PB for the adsorption of the studied PTEs, although slight differences were obtained: a higher adsorbed concentration of As was observed, resulting in higher adsorption at pH 8. The highest adsorbed concentrations occurred at alkaline pHs. Regarding P, no differences were observed with CB, even though the adsorbed concentration was 75 mmol/kg. However, a sudden decrease in P adsorption was observed at pH 7.4. This also coincided with the trends obtained by Romar-Gasalla et al. (2019) [50], although they found the lowest P adsorption at more acidic pHs. As regards F, adsorption was quite constant up to pH ≈ 8, resulting in ≈ 50 mmol/kg adsorbed. However, a slight increasing trend was observed, showing the maximum adsorption at pH 12 (57 mmol/kg). Additionally, in the case of CB, Cr adsorption was also highly affected by pH, resulting in pH-dependent behavior, showing the highest adsorption at pH ≈ 4 and the lowest at pH ≈ 10. These results were also obtained by Fiol et al. (2003) [26] using cork wastes, although the added concentration was 5-fold lower. Similar results were also found by Sfaksi et al. (2014) [27], who explained that a favored electrostatic attraction occurs between the highly protonated surface of CB and the predominant chromium species, promoting complexation phenomena at acidic pH. Although the trends were similar to those observed with CB, the adsorbed concentrations were slightly higher using PB than CB. Clearly, it is shown that the negative correlation between *K_F_* and pH is directly influenced by Cr behavior, whose adsorption sharply decreased with increasing pH using both biopowders. In the case of CB, this may be explained by a net negative charge on the surface of cork biomass with the increasing alkalinity of the solutions, as shown in Figure S1 (Supplementary Material), which is also in accord with Chubar et al. (2003) [57], while for the other elements, the influence of pH was not so relevant. The pH corresponding to the zero-point charge for CB was found to be 3.6 by Fiol and Villaescusa (2009) [58]. However, Castellar et al. (2019) [59] found a pH_ZPC_ for cork between 5.5 and 5.8, depending on the particle size. This means that at a pH higher than the pH_ZPC_, CB has a predominantly negative charge, and therefore, interactions with anionic species might be difficult. However, despite being the same product type, differences in composition may occur, leading to differences in the adsorption process [59]. Nonetheless, the presence of cations on the biopowder surface, together with factors such as hydration enthalpy, charge distribution, and ionic radius, may be the dominant factors in determining ion adsorption [60]. In the case of PB, the pH_ZPC_ was found to be 6.7 [61], 5.5 [62], and 3.8 [63], depending on the authors, indicating that pine bark bears a positive charge at a solution pH below pH_ZPC_. This explains the high Cr adsorption at acidic pH. However, no significant trends were found for the other PTEs regarding pH, meaning their different behaviors probably depend on the specific composition of the material, since pH_ZPC_ is highly variable. Similar results were also found for P [56].

## 4. Conclusions

The results obtained in the present study demonstrate that both CB and PB constitute highly effective bioadsorbent materials for the retention of potentially toxic elements such as P, As, F, and Cr, exhibiting high adsorption and simultaneously low desorption. The adsorption patterns varied depending on the type of PTE and the bioadsorbent material. Specifically, CB showed a higher capacity for As and F, while PB was more efficient for P and Cr. The adsorption curves followed the Freundlich and Langmuir models, confirming the surface heterogeneity and multilayer adsorption for most PTEs. Desorption tests revealed that both biopowders displayed low desorption rates, indicating high retention capacity. In this case, CB retained up to 99% of F and 85% of As, while PB showed retention rates up to 86% for Cr and 70% for P. For both studied biopowders, the influence of pH on the adsorption process was minimal for As, P, and F, but it significantly affected Cr adsorption, with higher adsorption at acidic pH values. Overall, these findings suggest that CB and PB biopowders are promising biosorbents for removing PTEs from aqueous solutions. Their effectiveness varies depending on the specific PTE and environmental conditions. The results underscore the potential of natural biopowders as eco-friendly alternatives for water treatment applications or soil solutions. Future work using these green materials could be focused on their addition to the soil as natural materials or even transformed into biochar for their use as both biosorbents for pollutants and soil carbon sequestrators. They can also be transformed into biochar and used in water for pollutant removal.

## Figures and Tables

**Table 1:** Physicochemical characteristics of both biopowders: cork bark and pine bark.

Material	Particle Size	Surface Area	pH_w_	pH_k_	C	N	C/N	Cellulose	Hemicellulose	Lignin	Rest *
		m^2^/g			----------%----------			-----------------------%------------------------			
CB *	Very fine (<100 µm)	0.57 ± 0.01	4.5 ± 0.1	3.4 ± 0.0	60.37 ± 0.32	0.60 ± 0.02	100.7	4.58 ± 0.03	7.45 ± 0.07	75.77 ± 0.19	12.2
PB *	Very fine (<100 µm)	0.36 ± 0.01	4.5 ± 0.1	4.0 ± 0.1	48.6 ± 2.00	0.08 ± 0.02	607.5	18.60 ± 0.10	14.70 ± 0.30	47.90 ± 0.03	4.1

* Data obtained by González-Feijoo et al. (2024) [28].

**Table 2:** Summary of adsorbed As, Cr, F, and P with different carbon-based materials.

Material	PTE Adsorbed (mmol/kg)				Range of Added Concentrations_mM	Reference
	As	Cr	F	P		
Cork bark powder	1022	668	1068	271	0.01–10	This study
Pine bark powder	455	720	471	556	0.01–10	This study
				0.37	0–0.5	Yeager and Wright (1982) [42]
		60	37		0.5–6	Romar-Gasalla et al., 2018 [41]
Hemp waste		9	16		0.5–6	Romar-Gasalla et al., 2018 [41]
Pine wood ash	13				0.01–1.33	Seco-Reigosa et al., 2013 [48]
Oak wood ash	11				0.01–1.33	Seco-Reigosa et al., 2013 [48]
		18	35		0.5–6	Romar-Gasalla et al., 2018 [41]
	55					Cela-Dablanca et al., 2022 [38]
			26.3		0.03–5.26	Quintáns-Fondo et al., 2016 [49]
Pine sawdust	7.5				0.01–1.33	Seco-Reigosa et al., 2013 [48]
				22.6	3.23	Romar-Gasalla et al., 2019 [50]
	1				0.1–1	Cela-Dablanca et al., 2022 [38]
			7.9		0.03–5.26	Quintáns-Fondo et al., 2016 [49]
Yohimbe bark		731			0.19–19.23	Fiol et al., 2003 [26]
Grape stalk		1058			0.19–19.23	Fiol et al., 2003 [26]
Olive stone wastes		154			0.19–19.23	Fiol et al., 2003 [26]
Cork wastes		288			0.19–19.23	Fiol et al., 2003 [26]
		5000.4			0.01–9.6	Sfaksi et al., 2014 [27]
	1.51				0.007–0.6	Almeida, A.D., 2015 [25]
Pretreated cork wastes	1.87				0.007–0.6	Almeida, A.D., 2015 [25]

**Table 3:** The maximum retention capacity of both biomaterials (CB and PB) tested for each PTE, expressed as a percentage. Data were calculated as the difference between maximum adsorption and maximum desorption values relative to the added PTE concentration.

PTE	Max Retention (%)	
	CB	PB
P	57	70
As	85	64
F	99	47
Cr	85	86

**Table 4:** Parameters corresponding to the adjustment of adsorption data to the Freundlich and Langmuir models for each PTE and for the two studied biopowder: cork bark and pine bark.

Biopowder		FREUNDLICH			LANGMUIR		
	PTE	K_F_	n	R^2^	K_L_	X_m_	R^2^
CB	P	80.1 ± 10.5	0.4 ± 0.1	0.881	1.71 ± 0.24	182.9 ± 7.6	0.991
	As	123,673.0 ± 6972.1	1.1 ± 0.0	1.000	-	-	-
	F	13,186.2 ± 674.9	1.1 ± 0.0	0.999	-	-	-
	Cr	294.9 ± 12.6	0.8 ± 0.0	0.992	0.13 ± 0.08	2559.4 ± 1278.0	0.987
PB	P	105.8 ± 8.1	1.3 ± 0.1	0.994	-	-	-
	As	266.7 ± 11.1	0.3 ± 0.0	0.984	1.95 ± 0.38	486.2 ± 25.2	0.986
	F	80.2 ± 6.5	1.0 ± 0.0	0.993	0.02 ± 0.02	5071.4 ± 4317.0	0.994
	Cr	427.5 ± 7.4	0.7 ± 0.0	0.997	0.48 ± 0.10	1393.3 ± 180.8	0.991

-: No fittings obtained. *K_F_*: Freundlich affinity coefficient (L*^n^* mmol^1−*n*^ kg^−1^); *n* (dimensionless): Freundlich linearity index; *X_m_*: maximum Langmuir adsorption capacity (mmol kg^−1^); *K_L_*: Langmuir constant related to the interaction intensity between adsorbent and adsorbate (L mmol^−1^); R^2^: adjusted coefficient of determination.

**Table 5:** Adsorption and desorption pH as a function of the added concentration for each PTE and biopowder studied: cork bark and pine bark.

PTE	Added Concentration (mM)	pH			
		CB		PB	
		ADS	DES	ADS	DES
P	0	5.3 ± 0.2	5.8 ± 0.0	4.8 ± 0.5	4.2 ± 0.1
	0.01	6.5 ± 0.1	5.7 ± 0.4	5.9 ± 0.1	3.9 ± 0.0
	0.025	6.3 ± 0.1	4.9 ± 0.1	6.0 ± 0.0	3.9 ± 0.1
	0.05	6.2 ± 0.0	5.0 ± 0.1	5.9 ± 0.0	3.9 ± 0.0
	0.1	6.3 ± 0.1	5.1 ± 0.0	5.9 ± 0.2	4.0 ± 0.0
	0.25	5.1 ± 0.1	5.9 ± 0.1	5.9 ± 0.1	4.0 ± 0.0
	0.5	5.1 ± 0.0	5.8 ± 0.0	5.6 ± 0.1	3.9 ± 0.1
	1	5.0 ± 0.1	5.6 ± 0.0	5.1 ± 0.0	3.9 ± 0.0
	2.5	5 ± 0.2	5.7 ± 0.1	4.9 ± 0.0	4.0 ± 0.0
	5	4.8 ± 0.1	5.6 ± 0.1	4.8 ± 0.2	4.0 ± 0.1
	10	4.7 ± 0.0	5.5 ± 0.0	4.5 ± 0.0	4.1 ± 0.0
As	0	3.9 ± 0.0	3.9 ± 0.1	5.1 ± 0.0	4.0 ± 0.0
	0.01	6.3 ± 0.1	5.4 ± 0.1	5.1 ± 0.0	4.2 ± 0.1
	0.025	6.5 ± 0.5	5.8 ± 0.9	5.0 ± 0.0	4.1 ± 0.0
	0.05	5.8 ± 0.5	5.4 ± 0.4	4.9 ± 0.0	4.2 ± 0.0
	0.1	6.1 ± 0.0	5.4 ± 0.5	5.0 ± 0.1	4.1 ± 0.0
	0.25	4.1 ± 0.0	4.0 ± 0.0	5.1 ± 0.1	4.2 ± 0.1
	0.5	4.4 ± 0.1	4.0 ± 0.1	5.2 ± 0.0	4.1 ± 0.1
	1	5.2 ± 0.1	3.9 ± 0.3	5.4 ± 0.0	4.0 ± 0.2
	2.5	6.3 ± 0.0	4.6 ± 0.8	6.4 ± 0.1	4.5 ± 0.0
	5	6.7 ± 0.0	5.6 ± 0.5	6.8 ± 0.1	4.6 ± 0.6
	10	7.0 ± 0.1	6.6 ± 0.0	7.1 ± 0.0	5.9 ± 0.2
F	0	3.6 ± 0.0	3.4 ± 0.0	4.9 ± 0.2	4.1 ± 0.3
	0.01	6.1 ± 0.0	5.9 ± 0.6	5.1 ± 0.0	3.9 ± 0.0
	0.025	5.8 ± 0.4	5.3 ± 0.2	5.2 ± 0.2	4.0 ± 0.1
	0.05	5.5 ± 0.4	4.9 ± 0.3	5.3 ± 0.1	3.9 ± 0.0
	0.1	5.4 ± 0.6	5.2 ± 0.7	5.2 ± 0.0	3.8 ± 0.1
	0.25	3.5 ± 0.0	3.4 ± 0.0	5.2 ± 0.0	4.0 ± 0.1
	0.5	3.5 ± 0.0	3.5 ± 0.0	5.4 ± 0.0	4.0 ± 0.1
	1	3.6 ± 0.0	3.4 ± 0.0	5.5 ± 0.6	4.0 ± 0.0
	2.5	3.7 ± 0.1	3.5 ± 0.0	4.5 ± 0.1	4.1 ± 0.0
	5	3.9 ± 0.0	3.6 ± 0.0	4.2 ± 0.0	4.2 ± 0.4
	10	4.1 ± 0.1	3.7 ± 0.1	4.1 ± 0.0	4.3 ± 0.1
Cr	0	4.4 ± 0.1	4.3 ± 0.1	5.1 ± 0.2	4.2 ± 0.0
	0.01	6.2 ± 0.1	5.2 ± 0.2	5.2 ± 0.0	4.3 ± 0.1
	0.025	6.2 ± 0.7	5.6 ± 0.6	5.4 ± 0.1	4.4 ± 0.1
	0.05	6.7 ± 0.0	6.0 ± 0.3	4.9 ± 0.4	4.4 ± 0.2
	0.1	6.6 ± 0.1	5.8 ± 0.8	5.0 ± 0.1	4.9 ± 0.0
	0.25	4.5 ± 0.0	4.3 ± 0.0	5.0 ± 0.2	4.4 ± 0.0
	0.5	4.8 ± 0.0	4.4 ± 0.0	4.7 ± 0.1	4.7 ± 0.2
	1	5.8 ± 0.0	4.4 ± 0.0	5.0 ± 0.1	4.3 ± 0.0
	2.5	6.3 ± 0.0	4.8 ± 0.2	5.7 ± 0.0	5.1 ± 0.1
	5	6.4 ± 0.0	5.6 ± 0.2	6.2 ± 0.1	4.2 ± 0.6
	10	6.3 ± 0.0	6.0 ± 0.1	6.2 ± 0.1	4.0 ± 0.1

## Data Availability

The data presented in this study are available on request from the corresponding author due to legal reasons.

## Supplementary Materials

The following supporting information can be downloaded at https://www.mdpi.com/article/10.3390/ma18030625/s1: Figure S1: Z potential of both studied materials at different pH; Table S1: Concentrations of the PTEs studied in the composition of the studied materials; Table S2: Functional groups of the studied material ‘surface. Figure S2: Carbon, Oxygen and Nitrogen functional groups detected in the surface material of CB (A–C) and PB (D–F), respectively. Each peak corresponds to an environment using the same transition or orbital. Reference [64] is cited in the supplementary materials.
