# Supplementary material for: Use of Biopowders as Adsorbents of Potentially Toxic Elements Present in Aqueous Solutions
Source: Materials (Basel). 2025 Jan 30;18(3):625. doi: 10.3390/ma18030625 (PMC11819779; doi:10.3390/ma18030625)
Supplement: Supplementary file 1 [file materials-18-00625-s001.zip › materials-3393754-supplementary.pdf]

Supplementary material:

Use of Biopowders as Adsorbents of Potentially Toxic Elements Present in Aqueous Solutions

Vanesa Santás-Miguel [1,2,3], Vanesa Lalín-Pousa [1,2], Manuel Conde-Cid [1,2], Andrés Rodríguez-Seijo [1,2] and Paula Pérez-Rodríguez [1,2,*]

[1] Department of Plant Biology and Soil Science, Area of Soil Science and Agricultural Chemistry, Faculty of Sciences, University of Vigo, 32004 Ourense, Spain; vsantas@uvigo.es (V.S.-M.); vanesa.lalin@uvigo.gal (V.L.-P.); manconde@uvigo.gal (M.C.-C.); andresrodriguezseijo@uvigo.gal (A.R.-S.)
[2] Agroecology and Food Institute (IAA), University of Vigo—Campus Auga, 32004 Ourense, Spain
[3] Microbial Ecology, Department of Biology, Lund University, Ecology Building, 22362 Lund, Sweden
* Correspondence: paulaperezr@uvigo.es

Figure S1

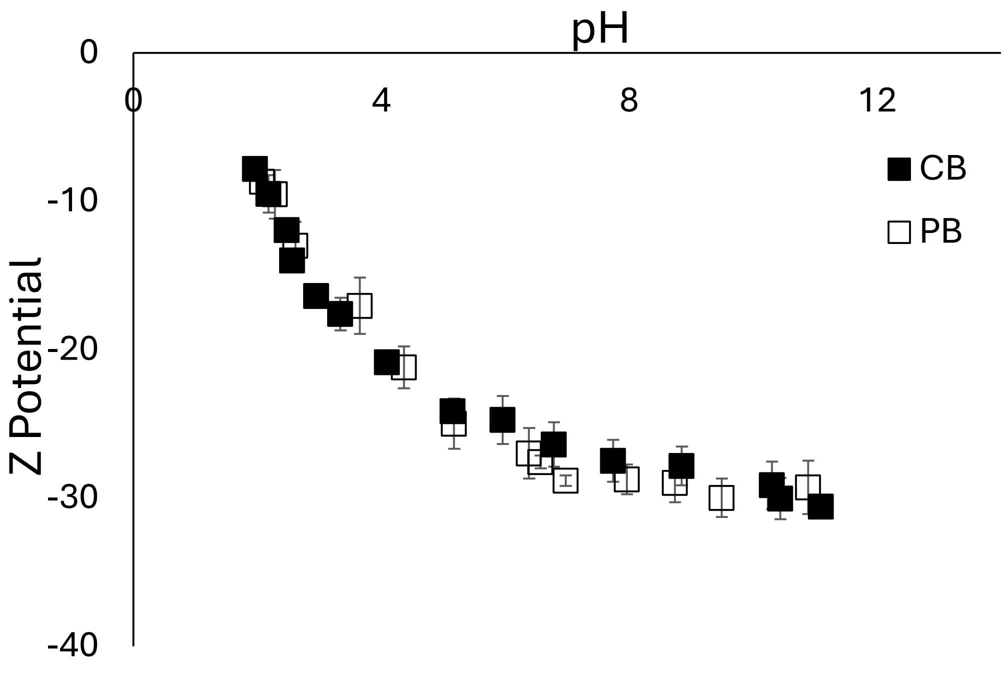

Figure S1. Z potential of both studied materials at different pH.

Table S1. Concentrations of the PTEs studied in the composition of the studied materials.

	**P**	**Cr**	**As**	**F**
	**mg/kg**			
**CB**	193.7	0.2	<LD	<LD
**PB***	<0.01	1.9	<0.001	<LD

*Data obtained from Rivas-Pérez et al., 2015.

Table S2. Functional groups of the studied material ʹsurface.

Biopowder	Element	Atomic %	C/O	Binding energy (eV)	Functional groups	Relative Atomic %
CB	C 1s	78.38	3.76	284.8	C1s 1 (C-C)	10.45
				286.47	C1s 2 (C-O, C-O-C)	46.05
				287.83	C1s 3 (C=O)	31.89
				289.33	C1s 4 (O-C=O)	11.61
	O 1s	20.86		531.3	Os1 1 (OH)	10.92
				533.45	Os1 2 (C-O-C, C=O, O-C=O)	55.22
				535.07	Os1 3 (Adsorbed H_2O)	33.86
	N 1s	0.75		400.28	N Organic	52.81
				401.74	Oxidized N	47.19
PB	C 1s	79.36	4.05	284.8	C1s 1 (C-C)	26.2
				286.32	C1s 2 (C-O, O-C-O)	20
				287.96	C1s 3 (C=O)	33.66
				289.5	C1s 4 (O-C=O)	14.45
				291.02	C1s 5 (CO_3)	5.7
	O 1s	19.58		532.01	Os1 1 (OH, C=O, O-C=O)	11.69
				533.65	Os1 2 (C-O-C)	32.37
				535.93	Os1 3 (Adsorbed H_2O)	55.94
	N 1s	1.06		ND	-	-

ND: Not detected, -: not applied. 1s correspond to the most intense and most likely orbital to be analyzed.

Figure S2

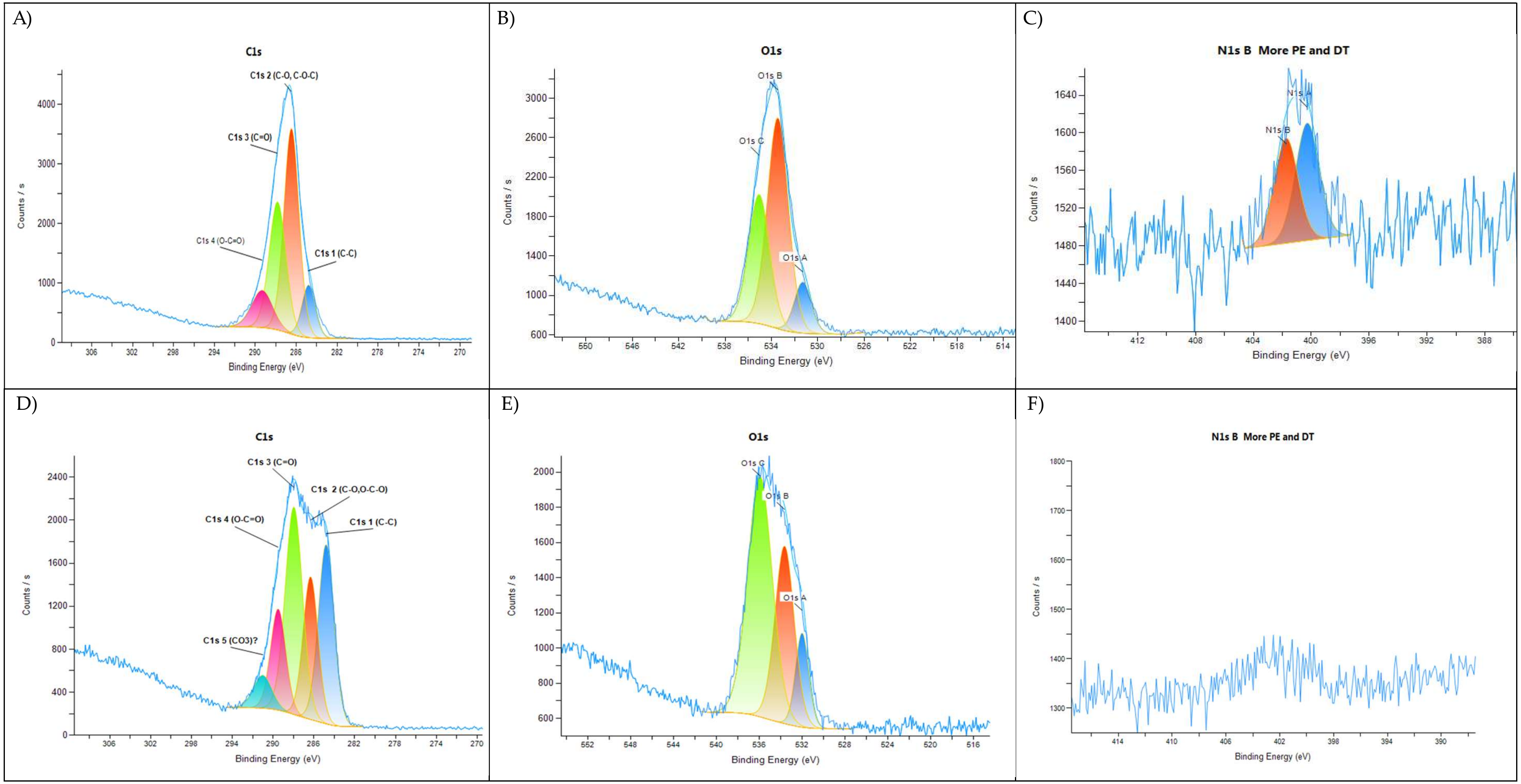

Figure S2. Carbon, Oxygen and Nitrogen functional groups detected in the surface material of CB (A-C) and PB (D-F), respectively. Each peak corresponds to an environment using the same transition or orbital.

References

[66] Rivas-Pérez, I.M.; Paradelo-Núñez, R.; Nóvoa-Muñoz, J.C.; Arias-Estévez, M.; Fernández-Sanjurjo, M.J.; Álvarez-Rodríguez, E.; Núñez-Delgado, A. As(V) and P Competitive Sorption on Soils, By-Products and Waste Materials. *Int. J. Environ. Res. Public Health* **2015**, *12*, 15706-15715. https://doi.org/10.3390/ijerph121215016
